# Comparative Flower Transcriptome Network Analysis Reveals *DEGs* Involved in Chickpea Reproductive Success during Salinity

**DOI:** 10.3390/plants11030434

**Published:** 2022-02-05

**Authors:** Mayank Kaashyap, Rebecca Ford, Anita Mann, Rajeev K. Varshney, Kadambot H. M. Siddique, Nitin Mantri

**Affiliations:** 1 The Pangenomics Group, School of Science, RMIT University, Melbourne 3083, Australia; mayank.kaashyap@rmit.edu.au; 2 School of Environment and Science, Griffith University, Nathan 4111, Australia; rebecca.ford@griffith.edu.au; 3 Division of Crop Improvement, ICAR-Central Soil Salinity Research Institute (CSSRI), Zarifa Farm, Karnal 132001, India; anita.mann@icar.gov.in; 4 Center of Excellence in Genomics & Systems Biology, International Crops Research Institute for the Semi-Arid Tropics (ICRISAT), Patancheru 502324, India; r.k.varshney@cgiar.org or; 5 The UWA Institute of Agriculture, The University of Western Australia, Perth 6001, Australia; kadambot.siddique@uwa.edu.au; 6 State Agricultural Biotechnology Centre, Centre for Crop and Food Innovation, Food Futures Institute, Murdoch University, Murdoch, WA 6150, Australia

**Keywords:** salinity, pollen tube, transcription factor, phytohormone signalling, ion-homeostasis, co-expression network

## Abstract

Salinity is increasingly becoming a significant problem for the most important yet intrinsically salt-sensitive grain legume chickpea. Chickpea is extremely sensitive to salinity during the reproductive phase. Therefore, it is essential to understand the molecular mechanisms by comparing the transcriptomic dynamics between the two contrasting genotypes in response to salt stress. Chickpea exhibits considerable genetic variation amongst improved cultivars, which show better yields in saline conditions but still need to be enhanced for sustainable crop production. Based on previous extensive multi-location physiological screening, two identified genotypes, JG11 (salt-tolerant) and ICCV2 (salt-sensitive), were subjected to salt stress to evaluate their phenological and transcriptional responses. RNA-Sequencing is a revolutionary tool that allows for comprehensive transcriptome profiling to identify genes and alleles associated with stress tolerance and sensitivity. After the first flowering, the whole flower from stress-tolerant and sensitive genotypes was collected. A total of ~300 million RNA-Seq reads were sequenced, resulting in 2022 differentially expressed genes (DEGs) in response to salt stress. Genes involved in flowering time such as *FLOWERING LOCUS T (FT)* and pollen development such as *ABORTED MICROSPORES (AMS),* rho-GTPase, and pollen-receptor kinase were significantly differentially regulated, suggesting their role in salt tolerance. In addition to this, we identify a suite of essential genes such as *MYB* proteins, *MADS*-box, and chloride ion channel genes, which are crucial regulators of transcriptional responses to salinity tolerance. The gene set enrichment analysis and functional annotation of these genes in flower development suggest that they can be potential candidates for chickpea crop improvement for salt tolerance.

## 1. Introduction

Chickpea is the most important cool-season food legume and provides nutritional food to the growing population [1,2]. With climate change, chickpea will become an increasingly important crop [3,4,5]. Over the years, chickpea production has increased from 6.4 to 14.7 million tons due to its growing demand and economic viability [6]. However, salinity is a significant constraint that reduces chickpea production by 8-10 per cent at a global scale— a static which is increasingly alarming in the context of growing population and climate challenged food security [7,8,9,10]. 

Chickpea is intrinsically sensitive to salinity during the reproductive stages, affecting the crop yields [1,11]. It can withstand salinity up to an electrical conductivity (EC) value of less than or equal to 1.0 dS/m compared to cereals, which can tolerate up to an EC equal to 10.0 dS/m [12]. Considerable breeding efforts have been made to underpin the salt tolerance traits; however, confounding effects of abiotic stress during the field trials and the polygenic nature of salt stress have rendered these approaches inadequate to uncover the intricate nature of complex gene networks. The QTLs identified for salt tolerance traits do not span the major and minor genes underlying the salt tolerance molecular mechanisms [13]. Furthermore, due to continued climatic shifts, these QTLs ought to be unstable and thus far could not be deployed in crop breeding programs to improve salt tolerance and develop new crop varieties. 

Flowering time plays a crucial role in crops’ adaptations and yield stabilisations in response to environmental cues [14,15,16]. Physiological studies have shown that salinity delays flowering time and severely affects the pod filling stages [9,17]. Despite pollen viability, sensitive genotypes show a higher occurrence of empty pods and seed abortions [12,18,19]. This observation suggests a failure in ovule fertilisation as the main reason for pod abortion or empty pods, despite the viable pollen and pollen tube growth. Additionally, chickpea has a narrow genetic base. It shows phenotypic plasticity, making it challenging to underpin the physiological responses and uncover the genes responsible for flower development during salt stress [20,21]. It is essential to understand the transcriptome dynamics and elucidate the molecular mechanisms in response to salt tolerance to unravel the phenotypic plasticity barriers.

Uncovering the molecular mechanisms and identifying potential candidate genes to overcome the phenotypic plasticity barriers would allow crop improvement, tapping into the genetic variation. Several studies reported that salt stress induces complex regulatory mechanisms and major transcriptional reorganisation [22,23,24,25]. These genes were differentially up- and down-regulated between the contrasting genotypes and developmental stages, and they encode for cell wall biogenesis, heat shock proteins, and transcription factors [26,27]. Despite considerable efforts, the cis-acting genes that regulate this multigenic trait and their complex interplay, which changes the function of genes upon interactions in a network, could not be intuitively elucidated till now. 

Transcriptomics has offered a deep understanding of gene regulation. Therefore, identifying the differentially regulated candidate genes from flowers of contrasting the tolerant and sensitive genotypes grown under saline conditions can shed light on salt tolerance mechanisms [28,29]. There are success stories on improving salt tolerance in crops such as wheat, rice, and soybean, where identification of potential candidates such as transmembrane ion-transporter proteins was reported to regulate cellular ion homeostasis. 

The ability of the chickpea genotype to maintain more seeds under salinity is a measure of crop salt tolerance [30]. Based on this trait, two contrasting salt responsive genotypes, JG11 and ICCV2, were selected from a chickpea mini-core collection and a reference set of diverse genotypes showing consistent salt tolerance variations in field trials [31]. These contrasting genotypes are parents of Recombinant Inbred Lines (RILs) mapping population segregating for salt tolerance. The study aims to compare the transcriptomes of these genotypes to understand the molecular mechanisms regulating salt tolerance and identify the minor and major genes underlying the QTLs identified in the RILs to overcome the phenotypic plasticity of chickpea to the environmental stress.

## 2. Results

### 2.1. Transcriptome Assembly 

**Figure 1:**
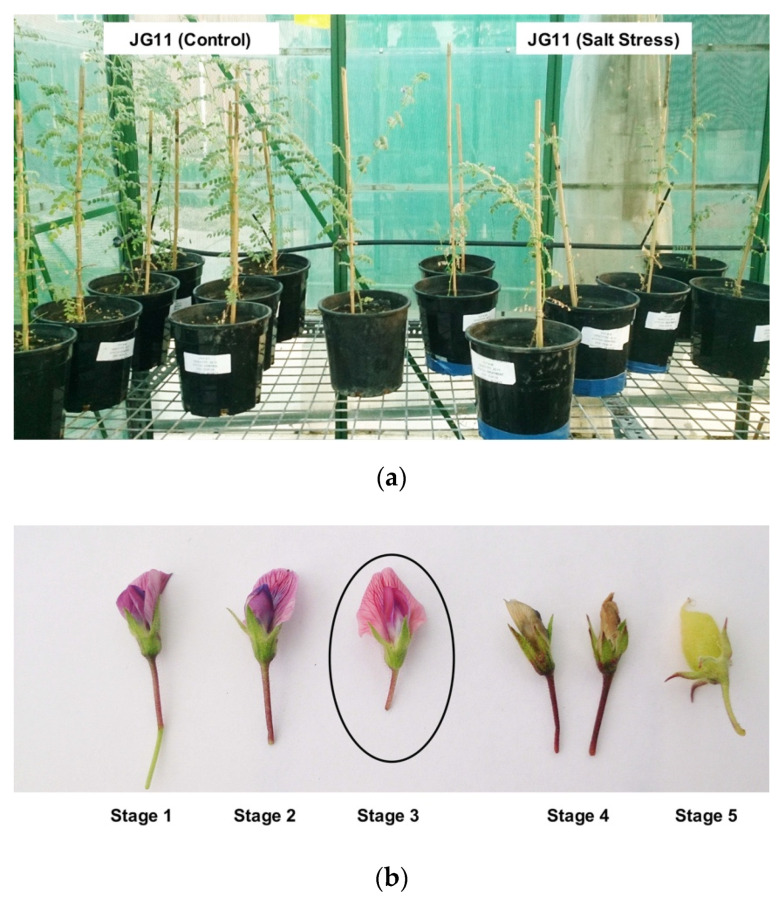
(**a**) Physiological responses of chickpea tolerant genotype (JG11) to salt stress in the glasshouse. A total of 80 mM NaCl was added and electrical conductivity (EC) was maintained at 1 dS/m as a measure of salinity throughout the experiment. The first flower was observed in JG11 after 34 days and ICCV2 after 30 days of sowing. As an effect of salt stress, plants show reduced plant height, slightly delayed flowering in the stressed tolerant plants, and fewer flowers in the sensitive genotype. (**b**) Flower developmental stages. Stage 3 fully opened flowers from both control and stress conditions were collected for transcriptomic analysis.

**Figure 2:**
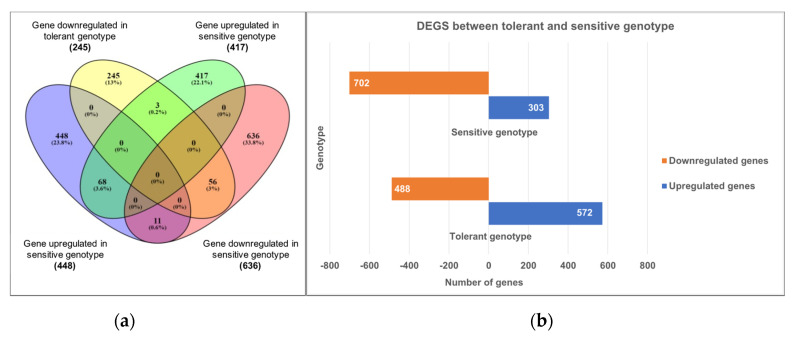
(**a**) Common and exclusive DEGs between the tolerant and sensitive genotypes. (**b**) The number of DEGs is significantly up- and down-regulated between the two genotypes.

A total of twelve flower transcriptomes from three biological replicates of JG11 and ICCV2 subjected to control and salt stress conditions were sequenced to ~50 million reads per sample (Figure 1). A reference guided transcriptome assembly was generated from the reads sequenced from two chickpea genotypes to study the differential transcriptomic regulation involved in reproductive success during salt stress. The reference guided assembly comprised 36,154 genes, which is 8.4% more than the currently reported genes in the chickpea genome (33,351), and additionally identified 3465 novel genes unannotated in the latest version [32]. A total of 2022 genes were differentially expressed between the flowers of the two contrasting genotypes in response to the salt stress compared against control conditions. The tolerant genotype had a more significant number of up-regulated (572) genes and a smaller number of down-regulated (303) genes, whereas the sensitive genotype had less up-regulated (488) and more down-regulated genes (702) (Figure 2). On comparing the commonalities between the differentially expressed genes (DEGs) expressed in the two genotypes, 448 genes were induced while 245 genes were repressed exclusively in the tolerant genotype. 

On the contrary, 417 genes were induced while 636 genes were repressed exclusively in the sensitive genotype. Interestingly, genes such as Cytochrome P450 and *MYB* transcription factor were commonly differentially expressed between the contrasting genotypes. However, these were induced in the tolerant genotype and repressed in the sensitive genotype.

#### 2.1.1. Gene Set Enrichment Analysis

**Figure 3:**
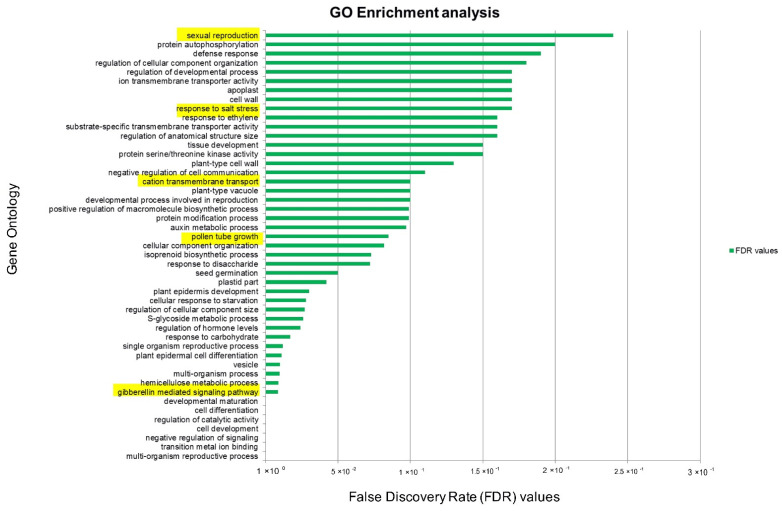
Gene set enrichment analysis shows enriched GO categories based on differentially expressed genes between the tolerant genotype and sensitive genotype flowers in response to salt stress.

The False Discovery Rate (FDR) values (0.05) of differentially expressed genes were converted to gene ID table and used for GSEA in Blast2GO software. On comparing DEGs from the tolerant against sensitive, the significant GO categories enriched were a response to salt stress (GO:0009651); gibberellin-mediated signalling pathway (GO:0009740); pollen tube development (GO:0009860); sexual reproduction (GO:0019953); transition metal ion binding (GO:0046914); and regulation of hormone level (GO:0010817) (Figure 3).

#### 2.1.2. Differentially Expressed Genes in Response to Salt Stress

**Figure 4:**
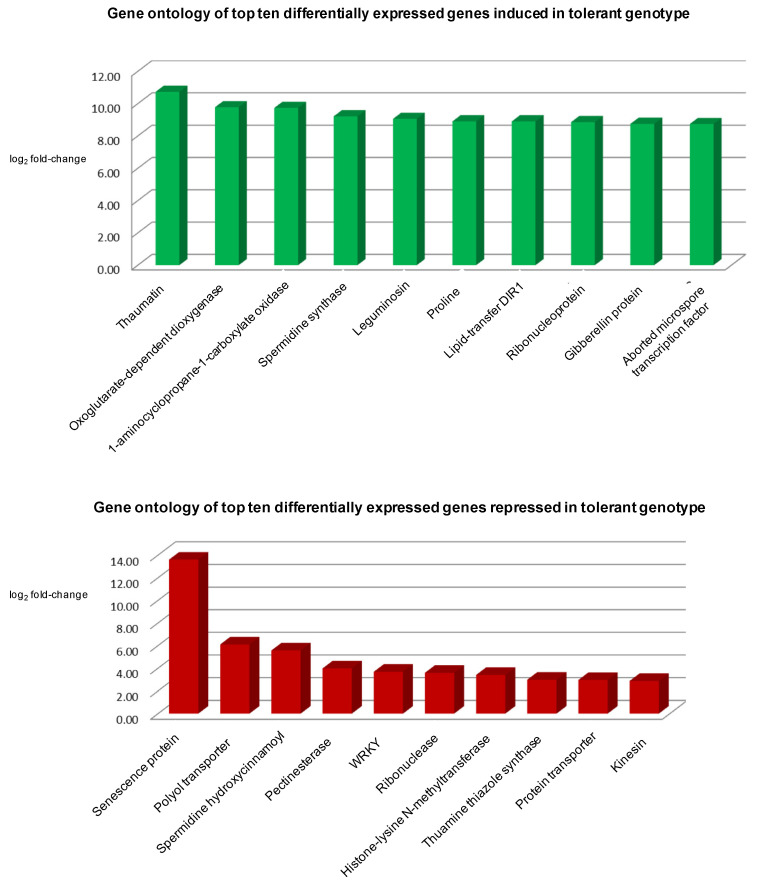
Top ten differentially expressed genes in tolerant genotype response to salt stress.

**Figure 5:**
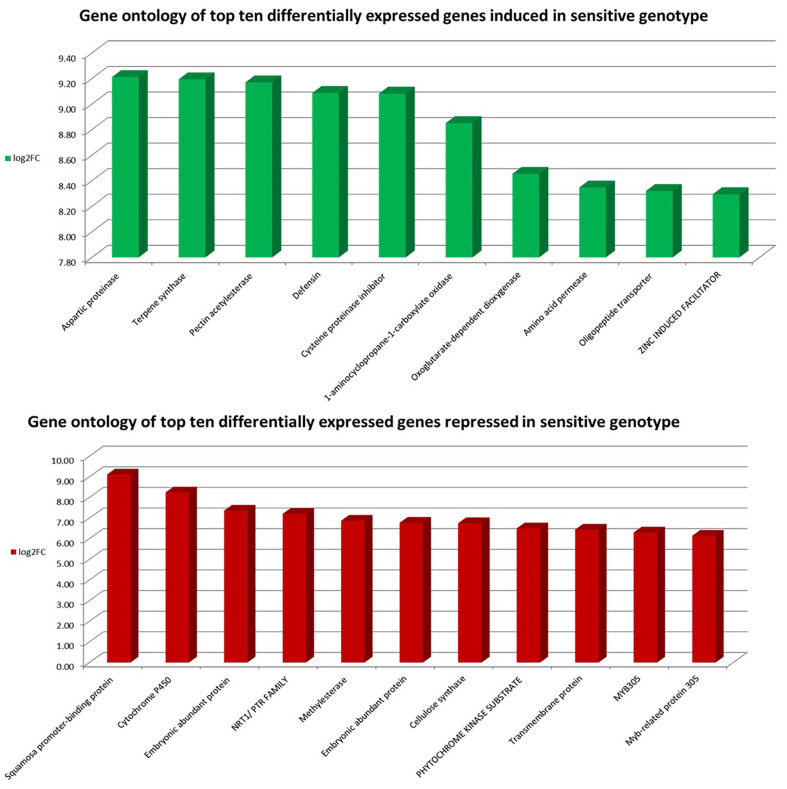
Top ten differentially expressed genes in sensitive genotype in response to salt stress.

The top differentially expressed genes in the tolerant genotype were thaumatin proteins, oxoglutarate-dependent dioxygenase, spermidine synthase, and aminocyclopropane-1-carboxylate oxidase. Thaumatin protein gene was highly induced in the tolerant genotype (Ca30893; FC: 1698.44 ↑) while repressed in the sensitive genotype (Ca30893; FC: −1.16 ↓). Thaumatin proteins are osmotins that belong to the pathogenesis-related (PR) genes. Thaumatin gene has been reported to be up-regulated in response to biotic and abiotic stresses [33]. Thaumatin is a stress-responsive gene that imparted salt tolerance in Ocimum and peanut [34,35]. Another vital gene, Enzyme inactive 2- (*AOP2*), was highly induced in both tolerant (Ca20688; FC: 873.0 ↑) and sensitive genotype (Ca06612; FC: 349.7 ↑). The *AOP2* has biochemical significance and is involved in the biosynthesis of phytochemicals such as flavonoids, gibberellins, and ethylene [36]. The genotypes significantly differentially regulated other essential salt stress-related genes such as jasmonic acid, gibberellin responsive, polygalacturonase, and pollen receptor (Figure 4 and Figure 5).

#### 2.1.3. Genes That May Determine Reproductive Success under Salt Stress

- Pollen tube development

**Figure 6:**
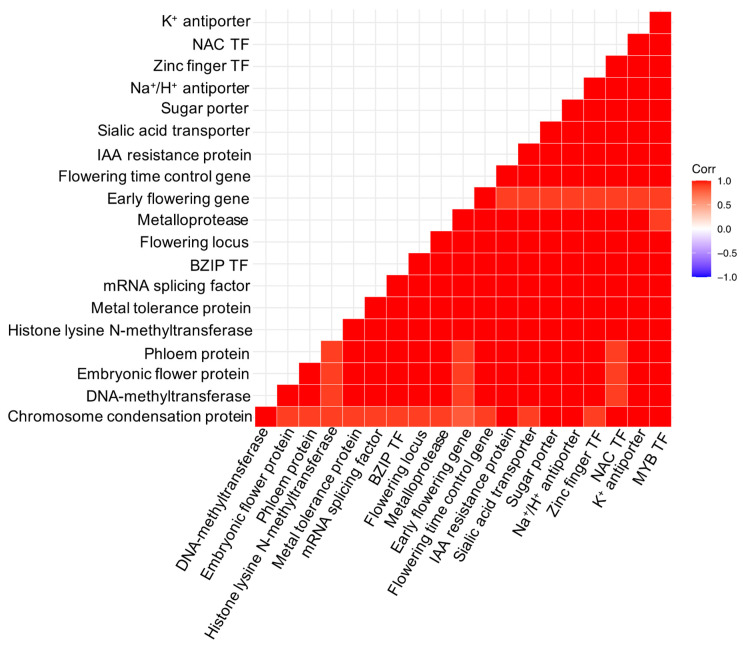
Correlogram showing co-expression of flowering locus gene with transcription factors and cell transporter during flower development in response to salt stress. Flower genes show high Pearson correlation coefficient values (red) with essential salt responsive genes.

Reproductive success measures crop yield that depends on the flower development and growth of the pollen tube, delivering male gametes to the ovary [37]. We identified a suite of genes that are thought to regulate flowering as an essential mechanism in response to salt stress. The important genes that control flowering time include flowering time control (*FTC*), early flowering, and histone modification genes. These genes were highly co-expressed with Na^+^/K^+^ ion channel, chromatin genes, *MYB*-TFs, and flowering locus (*FLC*) (Figure 6). The upregulation of these genes in the tolerant genotype and down-regulation in the sensitive genotype suggest their essential role in regulating flower development during salt stress. 

Interestingly, several highly induced differentially expressed genes showing strong co-expression with ‘hub genes’ such as *MYB* and *MADS*-box in the gene network are thought to be involved in pollen development. We identified genes such as Guanine-nucleotide exchange factor, rop-guanine, lectin receptor kinase, and pollen allergen, which play an essential role in controlling the polarised pollen tube development through rho-GTPase activation pathways. The rop guanine nucleotide exchange gene was significantly induced in the tolerant genotype (Ca05726; FC: 3.36 ↑) but repressed in the sensitive genotype (Ca18294; FC: −2.77 ↓). Similarly, pollen receptor-like kinase (Ca16817; FC: 2.36 ↑) was induced in the tolerant genotype but significantly repressed in the sensitive (Ca16817; FC: −4.19 ↓). Many studies have reported the active role of lectin receptor kinase in pollen development, pollen interaction with stigma, pollen rupture in the embryo sac, pollen tube, and ovule interaction and response to environmental stimuli [38,39]. In *Arabidopsis*, the expression of lectin receptor kinase was shown to influence pollen development, pollen sterility, and microsporogenesis [40,41]. Significantly, G-type lectin S-receptor-like serine/threonine-protein kinase was highly induced in the tolerant genotype (Ca17969; FC: 6.77 ↑) but repressed in the sensitive (Ca17969; FC: −1.16 ↓). As reported in an earlier physiology study, this gene may determine seed abortion and an increased number of empty pods in the sensitive chickpea [10]. 

Further, pollen allergen protein catalyses methionine formation and lignification of the cell wall. It is thought to be involved in recognising pollen-stigma, pollen tube-style, cell wall metabolism, and abiotic stress responses [42]. The upregulation of this protein is associated with anatomical changes and vessel development in tissues under salt stress [43]. Pollen allergen protein was 38-fold more induced in the tolerant genotype (Ca15856; FC: 152.2 ↑) compared to the sensitive (Ca15856; FC: 4.05 ↑). Another most critical factor for fertilisation is gamete fusion. It has now been studied that gamete adhesion and membrane adhesion are caused by fusion proteins such as *GAMETE EXPRESS* (*GX2*) [44,45]. This vital gene was highly repressed in the sensitive genotype (Ca31124; FC: −3.53 ↓), indicating possible failure of reproductive success and seed formation in the sensitive genotype.

**Figure 7:**
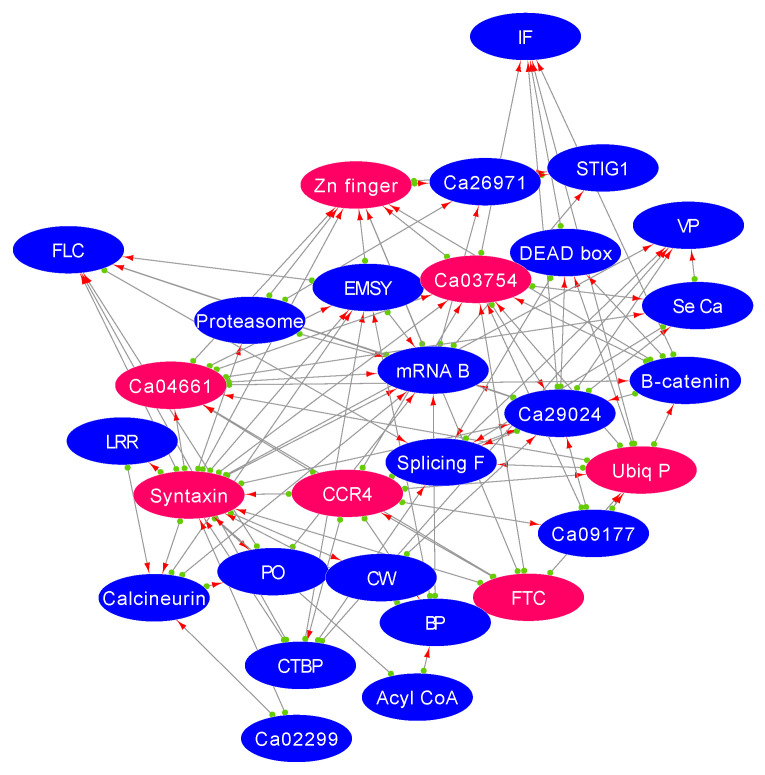
Weighted gene co-expression network analysis (WGCNA) shows gene signalling pathways involved in flowering time control response to salt stress. CW: cell wall genes; PO: polyamine oxidase; SeCa: serine carboxypeptidase; BP: mRNA binding protein, CCR4: CCR4-NOT transcription complex, IF: initiation factor; VP: vesicle protein, FTC: flowering time control; mRNA B: mRNA binding protein; CTBP: carboxy-terminal binding protein.

Furthermore, *STIG1* is an important gene that increases pollen tube growth. *STIG1* binds to pollen receptor kinase and has improved pollen tube growth and seed production [46]. *STIG1* was highly induced in the tolerant genotype (Ca07478; FC: 58.4 ↑) while repressed in the sensitive genotype (Ca07478; FC: −1.87 ↓) (Figure 7). The flowering time control (*FTC*) gene instigates an important gene involved in signalling, splicing, and transcription, leading to upregulation of the *FLC* and *STIG1* gene in the tolerant genotype. *FTC* gene significantly targets genes such as *CCR4*, syntaxin, Zn-finger TF, and ubiquitin-proteasome, suggesting that flower time is controlled through cell trafficking and homeostasis [47]. Membrane trafficking is often associated with floral organ separation and development. Modulations in these pathways could be an important mechanism that helps chickpea to maintain flower numbers during salt stress [48].

- Transcription factors (TFs) involved in flower development

A total of 183 transcription factor genes were differentially expressed, especially those specific to flower development. Out of these, 87 were up-regulated, while 101 were down-regulated. These families include *MYB* (25), *bZIP* (2), *bHLH* (24), *WRKY* (14), Zn-finger, *NAC* (5), ERF (18), TF-agamous MADS-box (8), HSF (6), TF-*CYCLOIDEA* (1), TF-*ABORTED MICROSPORES* (3), *GATA* (8), *GRAS* (3), trihelix (6), and *PLATZ* (2). Amongst these, *MYB*, agamous *MADS*-box, *bHLH*, *bZIP*, and *TF-ABORTED MICROSPORES* were induced while mainly *NAC* and *ERF* were repressed in the tolerant genotype (Table 1).

- MYB transcription factor may regulate salt stress response through agamous *MADS*-box cell dynamics

**Figure 8:**
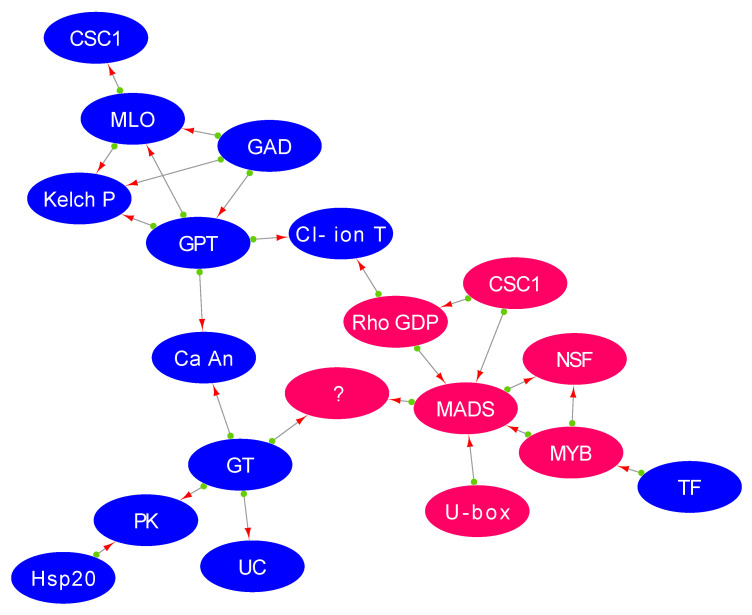
WGCNA gene regulatory network shows the ‘hub gene’ agamous *MADS*-box control the expression of genes involved in flower cell transport and development in response to salt stress. *MADS*: Agamous *MADS*-box; Ca An: α carbonic anhydrase; MLO: calmodulin binding defence response gene; NSF: N-ethylmaleimide sensitive factor; CSC1: Calcium permeable stress-gated cation channel 1; GT: glycosyltransferase; PK: protein kinase; Cl^−^ ion T: chloride channel; UC: UDP-glucuronic acid decarboxylase; GPT: glycerol-3-phosphate transporter; GAD: UDP-glucuronic acid decarboxylase; TF: truncated transcription factor; Rho GDP: Rho GDP dissociation inhibitor.

The two essential transcription factors, *MYB* and *AGAMOUS-MADS*-box protein, are thought to be widely distributed in plants. They regulate the elongation of stamen filament, anther development, pollen viability, pollen development, and flavonoid synthesis pathways [49,50]. Similarly, co-expression network analysis in our findings revealed that *MADS-box*—a target of the *MYB* gene—instigates the expression of genes involved in cell polarity and trafficking (Figure 8). The *MYB* transcription factor *MYB* 114, which is involved in pollen development, was induced in the tolerant genotype (Ca05149; FC: 5.24 ↑) while being highly repressed in the sensitive genotype (Ca01728; FC: −6.29 ↓). Interestingly, the upregulation of *MYB* induced *AGAMOUS-MADS*-box protein, which was 19-fold up-regulated in the tolerant genotype (Ca20056; FC: 76.10 ↑) compared to the sensitive genotype (Ca20056; FC: 4.08 ↑). The upregulation of this gene suggests floral and reproductive organ development, and cell division and enlargement in the tolerant genotype, as noted above during the salt stress.

Further, *MADS*-box gene targets the chloride ion channel, which is up-regulated in the tolerant genotype (Ca20075; FC: 5.93 ↑) and down-regulated in the sensitive genotype (Ca20075; FC: −1.18 ↓). This is an important salt tolerance mechanism, as chloride ions are more toxic for floral development during salinity. *MADS*-box triggered upregulation of the chloride transporter, which indicates an attempt to exclude these ions to avert flower abortion during salinity stress [51]. Further, this important signalling cascade coordinates the function of RhoGDP, which triggers the chloride ion channel [52]. Therefore, it may be hypothesised that the ‘hub gene’ MADS-box regulates the ion-exclusion mechanism to eliminate the toxic chloride ions promoting flower development in response to salt stress.

- AMS transcription factor may affect pollen development under salt stress

**Figure 9:**
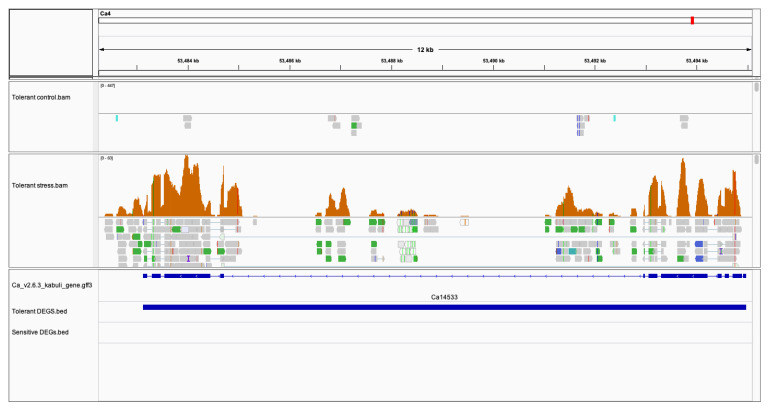
Integrative Genome Viewer (IGV) shows upregulation of *ABORTED MICROSPORES* (*AMS*) (Ca14533) gene in tolerant genotype as compared to sensitive genotype in response to salt stress. The tracks include CDC frontier Kabuli v2.6.3 reference fasta; annotated gene. gff3; accepted.hits.bam from tolerant control and control stress and differentially expressed gene coordinates.

The pollens are surrounded by a protective cell wall that provides resistance against environmental factors, desiccation, and pathogens, and helps pollen adhesion and development. Transcription factor *ABORTED MICROSPORES* plays a crucial role in tapetum development and is essential for male fertility and pollen differentiation within the developing anther [53]. Transcription factor *ABORTED MICROSPORES* (*AMS*) was highly induced in the tolerant genotype (Ca14533; FC: 427.5 ↑) while being repressed in the sensitive genotype (Ca14533; FC: −1.10 ↓) (Figure 9). This factor is reported to bind to the promoter of the genes responsible for pollen development, and its upregulation in the tolerant genotype suggests that this gene may be crucial for pollen viability during salt stress [54]. On the contrary, the repression of AMS genes in sensitive genotypes suggests a possible failure in anther and pollen development in the sensitive genotype. The inability of the pollen tube to develop and reach the ovary under salt stress conditions leads to flower abortion and low crop productivity in sensitive genotypes.

#### 2.1.4. Response of Hormone Signalling Genes

**Figure 10:**
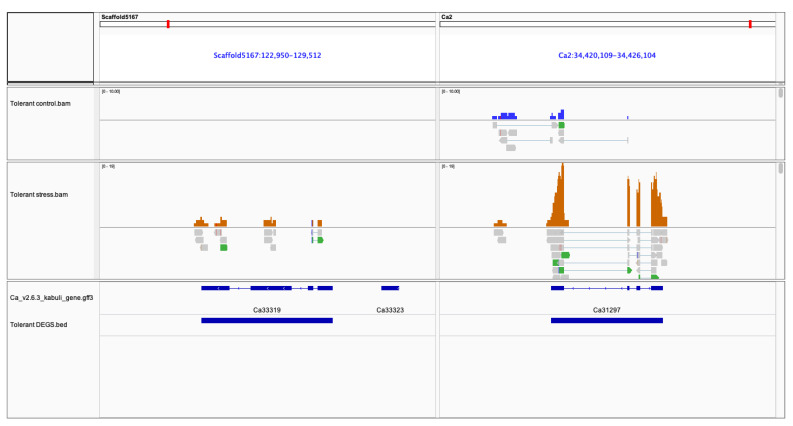
Integrative Genome Viewer (IGV) shows upregulation of jasmonic acid gene (Ca33319) induces *FLC* gene (Ca31297) in tolerant genotype in response to salt stress. The tracks include CDC frontier Kabuli v2.6.3 reference fasta; annotated gene. gff3; accepted.hits.bam from tolerant control and tolerant stress and differentially expressed gene coordinates.

Several studies have suggested that jasmonic acid mediates drought and salt stress response [55,56]. In our findings, the Jasmonic acid-amido synthetase (*JAR1*) gene was 15-fold up-regulated in the tolerant genotype (Ca33319; FC: 39.3 ↑) as compared to the sensitive (Ca33319; FC: 2.58 ↑) (Figure 10). The jasmonic acid-induced response is significant for pollen maturation; therefore, its upregulation suggests considerable involvement in stress management during reproductive processes [57].

**Figure 11:**
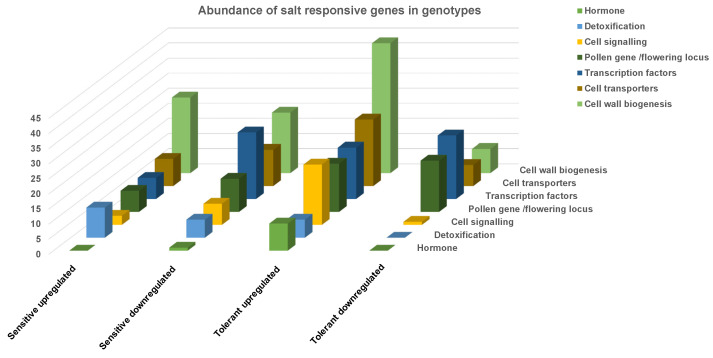
The abundance of gene families involved in hormone signalling and cell wall biogenesis in response to salt stress.

Ethylene is known as a stress hormone and its production increases in response to environmental stress. Aminocyclopropane-1-carboxylate oxidase is an essential precursor of ethylene, and its application on Arabidopsis has been shown to delay flowering. Several aminocyclopropane-1-carboxylate oxidase genes were 137-fold-induced in the tolerant genotype (Ca26892; FC: 849.2 ↑) compared to the sensitive genotype (Ca26892; FC: 6.23 ↑). This suggests that the first signalling response is ethylene production during the salt stress, which delays the flowering in the tolerant genotype. This could be an essential mechanism to escape salt stress and maintain more flowers. It has been reported earlier that salinity delays flowering time in chickpea [30], and the tolerant genotypes seem to employ this strategy to allow time for salt stress adaptation (Figure 11).

#### 2.1.5. Cell Wall Reorganisation May Be a Key Mechanism for Salt Tolerance

**Figure 12:**
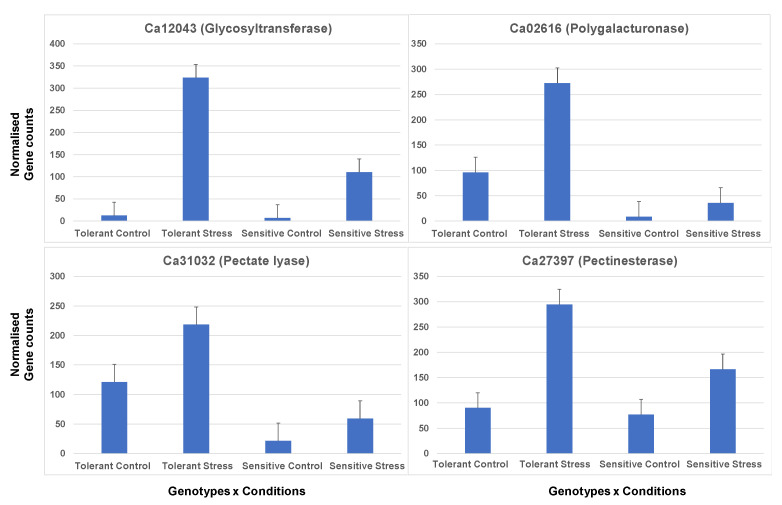
Differential expression of genes involved in cell wall biogenesis. Genes are induced in tolerant genotype stress conditions compared to sensitive genotypes in response to salt stress. The error bars are standard errors (SE) calculated using three biological replicates across the conditions.

Cell wall remodelling is essential in cell expansion, membrane transport, and stress signalling [58,59]. Several important genes involved in cell wall organisation such as polygalacturonase, pectinesterase, glycosyltransferase, and lipid transfer proteins were amongst the top differentially expressed genes having more significant fold change in the tolerant compared to the sensitive genotype. Glycosyltransferase overexpression in tobacco has been observed to enhance salt tolerance by increased accumulation of proline and sugars, and reduced Na+/K+ ion concentration [60,61]. We identified that glycosyltransferase genes were highly induced in the tolerant genotype (Ca12043; FC: 347.2 ↑) but were repressed in sensitive genotype (Ca00611; FC: −48.8 ↓). Notably, essential cell wall genes such as pectinesterase, polygalacturonase, and pectate lyase were highly induced during the salt stress in the tolerant genotype (Figure 12). The expression of these cell wall genes indicates the floral organs’ developmental process and the ion channels’ regulation. It could be that induction of these genes in the tolerant genotype denotes the elongation of the pollen tube during the salt stress.

#### 2.1.6. Role of Transporters in Ion-Homoeostasis 

Ion-exclusion is an essential mechanism for salt stress tolerance. Important ion channels such as potassium and chloride were significantly up-regulated in the tolerant genotypes but repressed in the sensitive genotype. Potassium ion channels exchange Na+ with K+ and regulate the ion-exclusion mechanism [62]. The potassium transporter gene was induced in the tolerant genotype (Ca29596; FC: 2.37 ↑) while repressed in the sensitive genotype (Ca21839; FC: −5.81 ↓). It is important to note that potassium ion uptake is also necessary for pollen development, and deficiency in K+ leads to infertility [63]. The repression of these transporter genes in the sensitive genotype confirms their incompetency to maintain cellular ion-homeostasis during the salt stress. This could have also affected the ability of the pollen tube to grow and lead the successful fertilisation. 

Micronutrients such as copper and boron play an essential role in pollen development, germination, and seed formation [64]. Plants maintain copper equilibrium below a toxic level through important transmembrane transporters called copper-transporting ATPase, and this gene was highly induced in tolerant genotype (Ca22802; FC: 50.9 ↑) while repressed in sensitive (Ca22802; FC: −3.22 ↓). Boron is an essential micronutrient, and upregulation of the boron transporter in the tolerant genotype (Ca13752; FC: 2.05 ↑), whilst being repressed in sensitive genotype (Ca13752; FC: −2.63 ↓), suggests boron uptake through the roots and xylem is utilised during the reproductive processes. These genes collectively regulate the ion-homeostasis, helping the flower development, and their upregulation in the tolerant genotype points to an essential molecular mechanism in response to salinity. 

### 2.2. Validation of RNA-seq Results with Quantitative Real-Time PCR (qRT-PCR)

**Figure 13:**
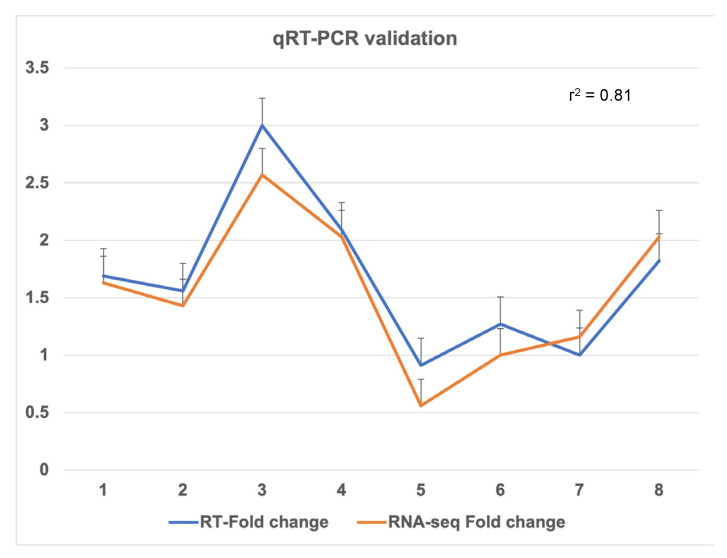
Validation of gene expression obtained from RNA-seq data using real-time quantitative PCR. A significant square of correlation value (r^2^ = 0.81) was obtained between the RNA-seq and qRT-PCR. The error bars are standard errors (SE) calculated using technical replicate Ct values. (Left to right) Genes on the graph show expression of sodium coupled neutral amino acid transporter 6, sodium transporter HKT, sodium coupled neutral amino acid transporter 4, folate biopterin transporter, sodium/pyruvate cotransporter (BASS2), sodium driven chloride bicarbonate exchanger, K(+) efflux antiporter 2, and chloride channel.

Furthermore, to confirm the normalised gene count values obtained from RNA-seq data, we performed qRT-PCR using ten salt responsive candidate genes. These genes majorly include candidate genes such as cation exchanger, blue copper, glutaredoxin, ascorbate oxidase, and chloride-channel protein. Fold changes obtained from qRT-PCR showed a significant square of correlation value (r^2^ > 0.81) with fold changes of cation exchanger, sodium-coupled amino acid transporter, and chloride channel obtained with RNA-seq data. Few genes showed fold-change less than RNA-seq data. However, they were similarly induced in the tolerant genotype while repressed in the sensitive genotype. These results confirmed the validity of RNA-seq data (Figure 13).

## 3. Materials and Methods

### 3.1. Plant Material and Experimental Design

A total of two chickpea genotypes, desi JG11 (salt-tolerant) and Kabuli ICCV2 (salt-sensitive), were subjected to salt stress in a random complete block design (RCBD) in the glasshouse at RMIT University, Australia [19]. The experiment comprised three biological replicates of each genotype subjected to control and salt stress conditions. Plants were cultivated in 10.5-inch diameter pots that weighed 9.5 kg of pasteurised sandy clay soil without added fertilisers. The soil was supplemented with a calculated *Rhizobium* culture to help plants establish symbiotic nitrogen fixation. Further, to avoid any salt leakage, the pots were sealed with sturdy tape. 

Based on the previous physiological studies, two adaptive salt doses of 40mM NaCl (~1.75 grams per kg of soil) were added to the soil, one at the sowing and another ten days after sowing time [31]. The dose of 80 mM NaCl equates to an EC value of 1 dS/m as a threshold for salt tolerance where plants could be challenged until maturity. After adding the second salt dose, the EC of the soil was measured and maintained at 1 dS/m over time. Seeds were soaked overnight and sprouted in Petri-dishes after the surface was sterilised with 70% EtOH, followed by rinsing twice with MilliQ water. Plants were routinely watered to the field capacity and fully open flowers at Stage 3 were collected for RNA isolation. 

### 3.2. RNA Isolation and Library Preparation

Total RNA was isolated using the Qiagen RNeasy kit (GmBH, Germany). The frozen flower tissues were ground to a fine powder and weighed to add calibrated volumes of lysis buffer. Finally, RNA was eluted in 60µl of RNase free water. Total RNA was quantified using Nanodrop (NanoDrop™ Lite Spectrophotometer, ThermoScientific) and qualitatively analysed on High Sensitivity RNA ScreenTape (Agilent 2200). Only RNA with RIN value > 7 was chosen to enrich mRNA.

### 3.3. mRNA Enrichment

From 1 µg of total RNA as starting material, poly(A+) was isolated using Dynabeads mRNA purification kit (Thermofisher Scientific, Carlsbad, CA, USA).

A total of twelve RNA-seq libraries were prepared using Truseq Stranded Total RNA kit (Illumina) from two genotypes at two conditions having three biological replicates each. A total of 100 ng of mRNA was fragmented, and first-strand, paired-end libraries (150 bp × 2) were generated following the steps of the standard protocol prescribed by Illumina. On average, ~50 million reads were generated per sample, with six samples per lane on HiSeq 3000. 

### 3.4. RNA-seq Data Analysis

The reads were checked for their quality, length distribution, and adapter contamination, using FastQC. The rRNA reads were filtered using SortMeRNA (sortmerna-intel/2.1), which has built rRNA databases [65]. The reads were trimmed to remove any adapter sequences using trimmomatic/0.36 and mapped to improved CDC frontier Kabuli v2.6.3 reference genome (http://doi.org/10.7946/P2G596, accessed on 12 December 2021) using tophat2 (tophat-gcc/2.0.13) [66]. Only the concordant pair alignments were accepted for further analysis. More than 97% of reads passed the rRNA filtering and QC-filtering, and 87% concordant pair alignment mapping rate to chickpea genome (v2.6.3) was observed. The accepted hits were used for gene counts using HTSeq (HTSeq-0.6.1p1) [67]. These gene counts for each replicate and condition were used to find the differentially expressed genes with EdgeR (GLM likelihood ratio test) using Blast2GO PRO software [68]. To see what genes and functions are enriched in response to salt stress, gene set enrichment analysis (GSEA) was performed using Blast2GO PRO software [69]. 

### 3.5. Gene Regulatory Networks

The gene regulatory networks were created using R Bioconductor packages ‘WGNCA’; ‘knitr’; ‘limma’; ‘ggplots’; and ‘reshape2′ [70,71]. The low gene counts were masked, and the remaining 18,654 genes were log-transformed. A similarity matrix was generated upon computing the correlation distance matrix using function cordist. An adjacency matrix was developed using the function adjacency.fromSimilarity with a power of 12, type = signed. We constructed a weighted network, and the threshold chosen to limit the number of edges was 0.999 and genes with edges lower than the threshold or with no edges were discarded. The non-positive and negative edges were screened and rescaled to ‘0′ and ‘1′. The adjacency matrix was converted to graphml format using package ‘igraph’ using the function graph.adjacency; weighted = TRUE; mode = undirected in R [72]. The graph was exported as graphml with a threshold correlation value between the nodes (representing the genes) greater than 0.80. The network of genes was visualised by exporting the graphml file in Cytoscape (v 3.8.2) [73]. Genes with a high degree of connectivity coefficient were the central ‘hub gene’ on the network. The network was visualised in Prefuse Force Directed layout in Cytoscape, and the edge weights were added to identify the source and targets gene. The correlogram was constructed using the ‘corrplot’ package, and the correlation matrix of the gene counts was calculated using the ‘cor()’ function in R programming. 

### 3.6. Real-Time Quantitative PCR (qRT-PCR)

We obtained the FASTA sequences of reported salt-tolerant candidate genes annotated in chickpea from the NCBI RefSeq [74]. qRT-PCR specific primer-pairs were designed from these candidate genes using Primer3 (v 0.4.0) and checked for their specificity using primer BLAST [75] (see Supplementary File). We used elongation factor (eEF-1α) and GAPDH as the reference gene and obtained the primer sequences reported in the previous study [76]. Total RNA isolated from the tolerant and sensitive flowers subjected to salt stress and control conditions was converted to cDNA using QuantiTect Reverse Transcription Kit (Qiagen, GmbH, Germany). The cDNA was amplified using the reference and candidate gene primers and QuantiFast SYBR^®^ Green PCR Kit (Qiagen, GmbH, Germany). The cycle of threshold (Ct) values obtained from three technical replicates of control and stress samples were averaged and subtracted from the reference gene Ct values. The fold-change of candidate genes was estimated by comparing the Ct values of stress samples against control samples using ∆∆Ct method [77]. To estimate the variance, fold-changes obtained from the qRT-PCR were plotted against those obtained from RNA-seq data to calculate the square of correlation (r^2^). 

## 4. Conclusions

The deep-sequenced transcriptomes of two contrasting salt responsive chickpea genotypes uncover the important molecular mechanisms regulating salt stress tolerance. The essential salt tolerance candidate genes are highly induced in the tolerant genotype but are repressed in the sensitive genotype, suggesting their potential role in regulating the tolerance to salinity. Co-expression network analysis reveals the cascade of genes involved in salt tolerance. Most of the differentially expressed genes have an essential role in pollen tube development, confirming the pollen tube’s inability to develop and reach the ovary under salt stress conditions, which leads to flower abortion and low crop productivity. The induction of pollen development genes in flowers of the tolerant genotype promotes successful reproduction and restores crop productivity under stress conditions. The comprehensive dissection of contrasting salt informed flower transcriptomes would provide important gene information to screen the RILs variants and facilitate further introgression of salt tolerance in early maturing and high yielding yet salt-sensitive chickpea genotypes. The deep-sequenced transcriptome data will assist users to mine the gene of their interest and its applicability should extend to other crops to improve salt tolerance.

## Figures and Tables

**Table 1:** List of the important differentially expressed genes in flowers of the tolerant and the sensitive genotype in response to the salt stress.

Gene ID	Gene Name	Tolerant Genotype (Fold Change)	Sensitive Genotype (Fold Change)
Ca30893	Thaumatin protein	1448.2	−1.3
Ca05548	EIN1	6.1	−12.1
Ca01237	Auxin Efflux Carrier	2.6	−36.8
Ca25222	Auxin transporters	3.2	−14.9
Ca12043	Glycosyltransferase	337.8	−48.5
Ca14828	AT5PTase	3.2	−29.9
Ca31090	Peroxidase	3.5	−13.9
Ca27453	Expansins	294.1	−1.9
Ca33278	Xyloglucan endotransglucosylase/hydrolase	3.2	−27.9
Ca14533	Transcription factor AMS	415.9	−1.1
Ca02821	bHLH79	4.9	−19.7
Ca09486	Cytochrome P450	97.0	−337.8
Ca13032	Squamosa promoter binding protein	3.5	−548.7
Ca25411	WRKY 75	2.8	−1.2
Ca05149	MYB 114	5.3	−6.5
Ca11519	WIP6	1.2	−36.8
Ca06632	Chalcone synthase	2.5	−1.1
Ca10483	Uridine 5’’’’monophosphate synthase	194.0	−0.9
Ca33071	Sucrose transport protein	18.4	−5.7
Ca05726	Rop guanine	3.2	−2.8
Ca16817	Pollen receptor−like kinase	2.3	−4.3
Ca17969	Gtype lectin Sreceptor	7.0	−1.1
Ca07478	STIG1	59.7	−1.9
Ca29596	Potassium transporter	2.3	−5.7
Ca21157	Spermidine synthase	256.0	−3.0
Ca14413	NRT1/PTR	97.0	−97.0
Ca18241	WAT1	3.7	−11.3
Ca15101	Nramp3	3.2	−9.8
Ca30961	Purple acid phosphatase	3.0	−1.1
Ca10443	Serine/threonine phosphatase	20.2	−3.68
Ca04326	High mobility group protein	59.3	−3.22
Ca04967	Cucumusin	25.9	6.4
Ca14115	Polygalacturonase	3.22	−14.5
Ca11519	WIP6	1.25	−35.5
Ca20075	Glutathione S-transferase/chloride channel	5.93	−1.18
Ca30961	purple acid phosphatase	3.11	−1.15
Ca01426	Nucleoporin	45.8	−1.80

## Data Availability

Data will be uploaded to the NCBI GEO database. Please contact the corresponding author.

## Supplementary Materials

The following are available online at https://www.mdpi.com/article/10.3390/plants11030434/s1. File: qRT-PCR specific primer-pairs.
